# MicroRNA 27b promotes cardiac fibrosis by targeting the FBW7/Snail pathway

**DOI:** 10.18632/aging.102465

**Published:** 2019-12-23

**Authors:** Qiang Fu, Zhihong Lu, Xiao Fu, Shitang Ma, Xiaochun Lu

**Affiliations:** 1 Department of Cardiovascular Surgery, The General Hospital of Tianjin Medical University, Tianjin, China; 2 Department of Anatomy and Histology and Embryology, School of Basic Medical Sciences, Tianjin Medical University, Tianjin, China; 3 Department of Biochemistry and Molecular Biology, School of Basic Medical Sciences, Tianjin Medical University, Tianjin, China; 4 College of Life and Health Sciences, Anhui Science and Technology University, Chuzhou, Anhui, China; 5 Department of Cardiology, The 2nd Medical Centre, PLA General Hospital, Beijing, China

**Keywords:** microRNA 27b, cardiac fibrosis, FBW7, Snail

## Abstract

Our study aspires to understand the impact of miR-27b on myocardial fibrosis as well as its functional mechanism. 12 days post the ligation of coronary artery in rats, the expression of miR-27b in the peri-infarction region was elevated. Treating cultivated rat neonatal cardiac fibroblasts (CFs) with angiotensin II (AngII) also enhanced the miR-27b expression. Forced expression of miR-27b promoted the proliferation and collagen production in rat neonatal CFs, as revealed by cell counting, MTT assay, and quantitative reverse transcription-polymerase chain reaction. FBW7 was found to be the miR-27b’s target since the overexpression of miR-27b reduced the transcriptional level of FBW7. The enhanced expression of FBW7 protein abrogated the effects of miR-27b in cultured CFs, while the siRNA silence of FBW7 promoted the pro-fibrosis activity of AngII. As to the mechanism, we found that the expression of FBW7 led to the degradation of Snail, which is an important regulator of cardiac epithelial-mesenchymal transitions. Importantly, inhibition of miR-27b abrogated the coronary artery ligation (CAL) induced cardiac fibrosis *in vivo*, suggesting that it might be a potential target for the treatment of fibrosis associated cardiac diseases.

## INTRODUCTION

The fibrotic formation is an important pathological characteristic linked to many types of cardiac disorders, such as myocardial infarction, myocardial ischemia, hypertrophic cardiomyopathies, and cardiac failure [1]. The fundamental of fibrotic formation is the adverse collection of collagen and extracellular matrix (ECM) proteins, which damage normal cardiac function and cause arrhythmia [2]. The fibrotic ECM leads to elevated rigidity and triggers pathological signals in cardiocytes, which result in heart failure. Fibroblasts primarily cause the deposition of the extra fibrotic ECM, and upon activation they directly result in hypertrophic cardiomyocytes through paracrine principles, aggravating the impairment in heart functioning [3, 4]. Fibroblasts in cardiac fibrosis are thought to be derived from epithelial-mesenchymal transition (EMT) of the epicardium-derived cells (EPDC) and promote their differentiation into fibroblast phenotype under the influence of growth factors, including TGF-β [5, 6]. Coupling of TGF-β with its cell-surface receptors signals, the Smad pathway mediates the transcriptional processes of some central fibrosis genes, such as fibronectin, collagens [7]. Although the use of TGF-β as a molecular target has been seen as a promising anti-fibrosis treatment, its impact on the cardiovascular system is ambiguous. Therefore, more efforts should be made to identify novel therapeutic targets for cardiac fibrosis.

MicroRNAs (miRNAs/miRs) have recently been under the spotlight to be one of the influential factors mediating gene expression, and essentially have an impact on the pathogenic mechanism underlying various diseases, including myocardial fibrosis [6, 8]. The miRNAs are highly conserved small noncoding RNAs that enable gene silencing by post-transcriptional mRNA degradation and suppression of the expressed protein [9]. miRNAs are formed in a strictly regulated process within the nucleus and are subsequently moved to the cell cytoplasm for processing the next step [9]. Several studies have indicated the significant contribution of miRNAs during the initiation and progression of diseases by regulating certain signaling pathways. The up-regulation of miR-21 stimulates the development of fibrosis [8], while enhanced overexpression of miR-29, miR-30, miR-133, or miR-590 [10] restrained the fibrotic reactions of cardiac fibroblasts (CFs). A recent study showed that overexpression of miR-27b in cardiocytes triggers cardiac hypertrophy and other disorders in animals [11]. However, the role and underlying mechanism for the infarction induced and miR-27b mediated cardiac fibrosis remains obscure. Moreover, a recent study reported that miR-27b also takes effects in mediating cancer cell EMT, and promotes cancer invasion and metastasis [12]. We, therefore, hypothesized that miR-27b might also promote EMT in cardiac fibrosis.

Our experiments investigated the cardiac infarction induced fibrosis, and found the capability of miR-27b, which is a frequently upregulated miRNA in hypertrophic and postinfarct hearts, to promote the CF generation in rats. We also observed that miR-27b inhibition by adenovirus transfection alleviated cardiac interstitial fibrosis and improved left ventricular compliance of rats subjected to coronary artery ligation. Our research suggested that manipulating the expression of miR-27b could be a potential treatment direction towards curing heart fibrotic disease.

## RESULTS

### MiR-27b increases in cardiac fibrosis patients and infarcted heart of rats

**Figure 1:**
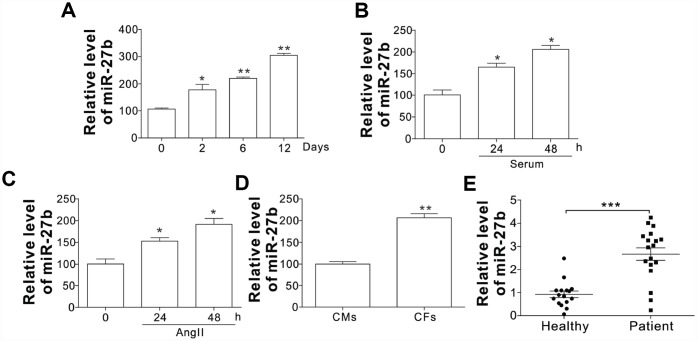
The miR-27b expression (**A**–**C**) The miR-27b expression in PIA of infarcted heart at different time points (**A**) and 10 % serum-treated CFs of rats (**B**) or angiotensin II (AngII, 100 nM; **C**) at designated time points. (**D**) The miR-27b expression in CMs and CFs. Data were represented as mean ± SEM (n=4). (**E**) The miR-27b expression in plasma specimens of healthy participants (n=16) and cardiac fibrosis patients (n=19). *, *p*<0.05; **, *p*<0.01; ***, *p*<0.001.

To investigate the role of miR-27 in the cardiac fibrosis, its expression in rat post-infarct cardiac tissues and cultured CFs was first detected under the conditions with significant CF proliferation. At 2, 6, and 12 d post-coronary artery ligation (CAL), the miR-27 expression was found to be elevated in the peri-infarct area (PIA) of rat heart compared to that of the control/sham-operated animals, as shown in Figure 1A. In contrast to the cells of the control group, the CFs from the neonatal rats after serum treatment showed increased miR-27 expression, as shown in Figure 1B. Additionally, Figure 1C reveals that miR-27b expression was enhanced after treating CFs with AngII (100 nM), a commonly used stimulus to induced cardiac fibrosis [13]. Besides, the CFs exhibited about 2-fold higher miR-27b expression relative to the cardiac monocytes (CMs) (Figure 1D). Furthermore, serum or AngII treatment did not significantly increase the miR-27b level in CMs (Supplementary Figure 1A, 1B), suggesting the specific role of miR-27b in CFs. To further confirm the regulation of miR-27b, we study its expression in acute ischemic stroke patients. Compared to the healthy controls, the acute ischemic stroke patients showed an expected rise in the miR-27b level in their circulating plasma (Figure 1E). Therefore, miR-27b was presumed to be involved in cardiac fibrosis.

### MiR-27b promotes CF proliferation

**Figure 2:**
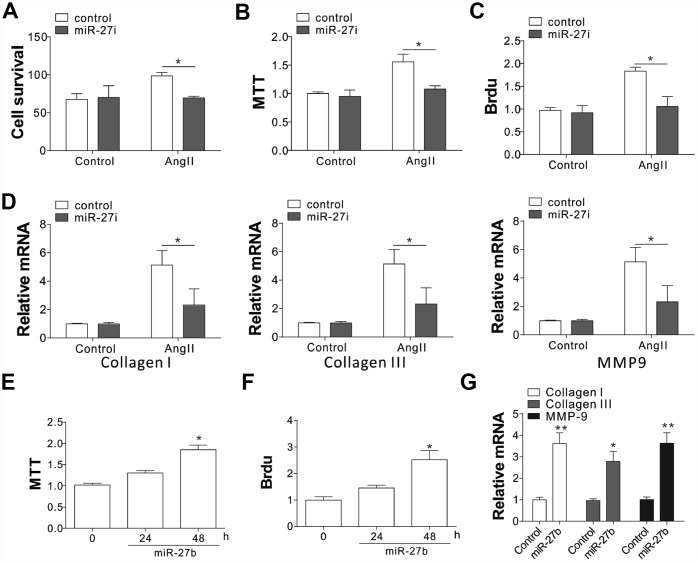
miR-27b expression promoted cardiac fibrosis (**A**–**C**) Supplementation of antagomiR-27b (miR-27i) inhibited the CF proliferation in neonatal rats. (**A**) Counting of cell numbers. (**B**) MTT analysis. (**C**) BrdU assay. (**D**) Collagen I, III, and MMP-9 mRNA levels in CFs treated with AngII in combination with miR27i. (**E**, **F**) Supplementation of miR-27b stimulated the proliferation of neonatal rats CF. (**E**) MTT test. (**F**) BrdU assay. (**G**) Collagen I, III, and MMP-9 mRNA levels in miR-27b-treated CFs. Data were represented as mean ± SEM (n=4). *, *p*<0.05; **, *p*<0.01.

To evaluate the role of miR-27b in cardiac fibrosis, the effect of miR-27b on neonatal CFs proliferation was evaluated by cell counting and MTT assay. The proliferation of CFs showed a steep rise upon treatment with AngII (100 nmol/L), as shown in Figure 2A–2C. Transfection of the miR-27 antagonist (miR-27i, 50 nmol/L) eliminated the AngII induced CF proliferation, working as miR-27’s specific inhibitor (Figure 2A–2C). Similarly, mRNA expression of collagen I, collagen III, and pro-matrix metalloproteinase (MMP)-9 increased after treatment with 100 nmol/L of AngII (Figure 2D), which was abolished after transfecting the cells with 50 nmol/L miR-27i (Figure 2D). Then transfection with miR-27b was performed to study its role in the proliferation of CFs. The results showed that both the growth and proliferation were promoted by the enhanced miR-27b expression (Figure 2E, 2F). Accordingly, collagen I, collagen III, and MMP-9 showed higher mRNA levels upon enhanced miR-27b expression (Figure 2G). Therefore, miR-27 was presumed to have a key impact on the CF cell proliferation and ECM expression.

### The miR-27b stimulates the CF proliferation by the induction of Snail

**Figure 3:**
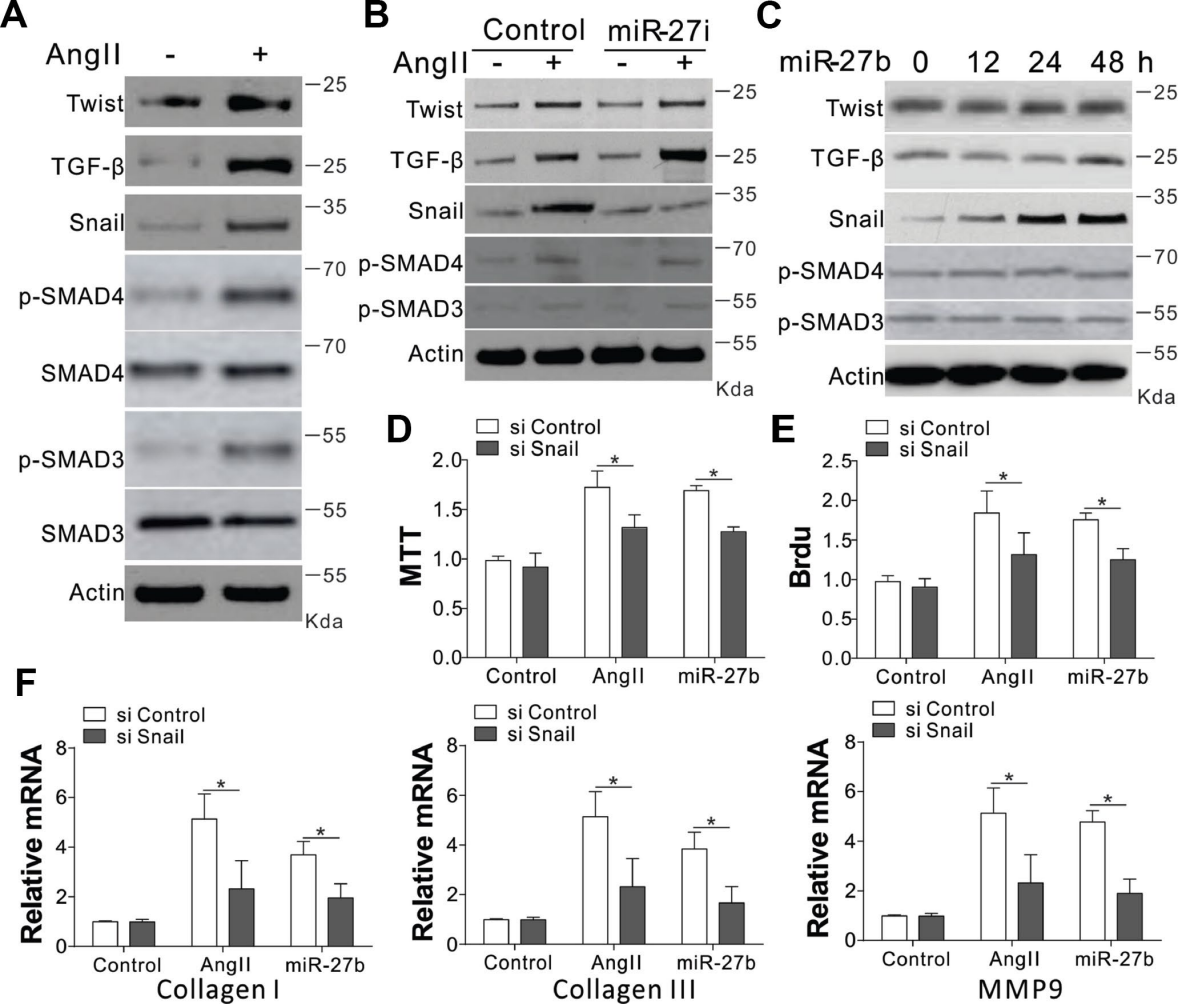
**Snail mediated proliferative effects of miR-27b on CFs.** (**A**–**C**) TGF-β, Twist, SMAD3/4, p-SMAD3/4, and Snail expression levels in CFs treated with AngII (**A**), AngII combined with miR-27i (**B**), and miR-27b (**C**). (**D**, **E**) The effect of silencing Snail on CFs cell proliferation induced by AngII or miR-27b was analyzed by MTT test (**D**) and BrdU assay (**E**). (**F**) Collagen I, III, and MMP-9’s mRNA levels in CFs subjected to treatment in (**D**). Data were represented as mean ± SEM (n=4). *, *p*<0.05.

The collagen I and III, and MMP-9 are reported to have crucial modulatory effects on cardiac fibrosis. We investigated the expression of their upstream factors, including Twist, TGF-β, SMAD3/4, and Snail, in CFs upon AngII treatment [7, 14]. Our results showed that AngII treatment in CFs induced the expression of Twist, TGF-β, and Snail, as well as the phosphorylation of SMAD3/4 (Figure 3A, Supplementary Figure 2A). However, miR-27b antagonist treatment specifically suppressed the induction of Snail, without influencing the AngII-induced TGF-β, Twist expression, or phosphorylation of SMAD3/4 (Figure 3B, Supplementary Figure 2B). It was further demonstrated that miR-27b transfection in CFs specifically induced Snail, and not Twist, TGF- β, p-SMAD3/4 (Figure 3C, Supplementary Figure 2C). These results suggest that the expression of Snail might contribute to miR-27b mediated cardiac fibrosis. To validate this hypothesis, the CFs were transfected with Snail siRNA. We found that depletion of Snail suppressed the cell growth and proliferation induced by AngII or miR-27b (Figure 3D, 3E). Moreover, the absence of Snail also abrogated the expression of collagen I and III, and MMP-9 induced by AngII or miR-27b in CFs (Figure 3F). Therefore, our results suggest that Snail mediated the proliferative effect of miR-27b as well as the cellular matrix growth in CFs.

### The miR-27b enhances the expression of Snail by targeting FBW7

**Figure 4:**
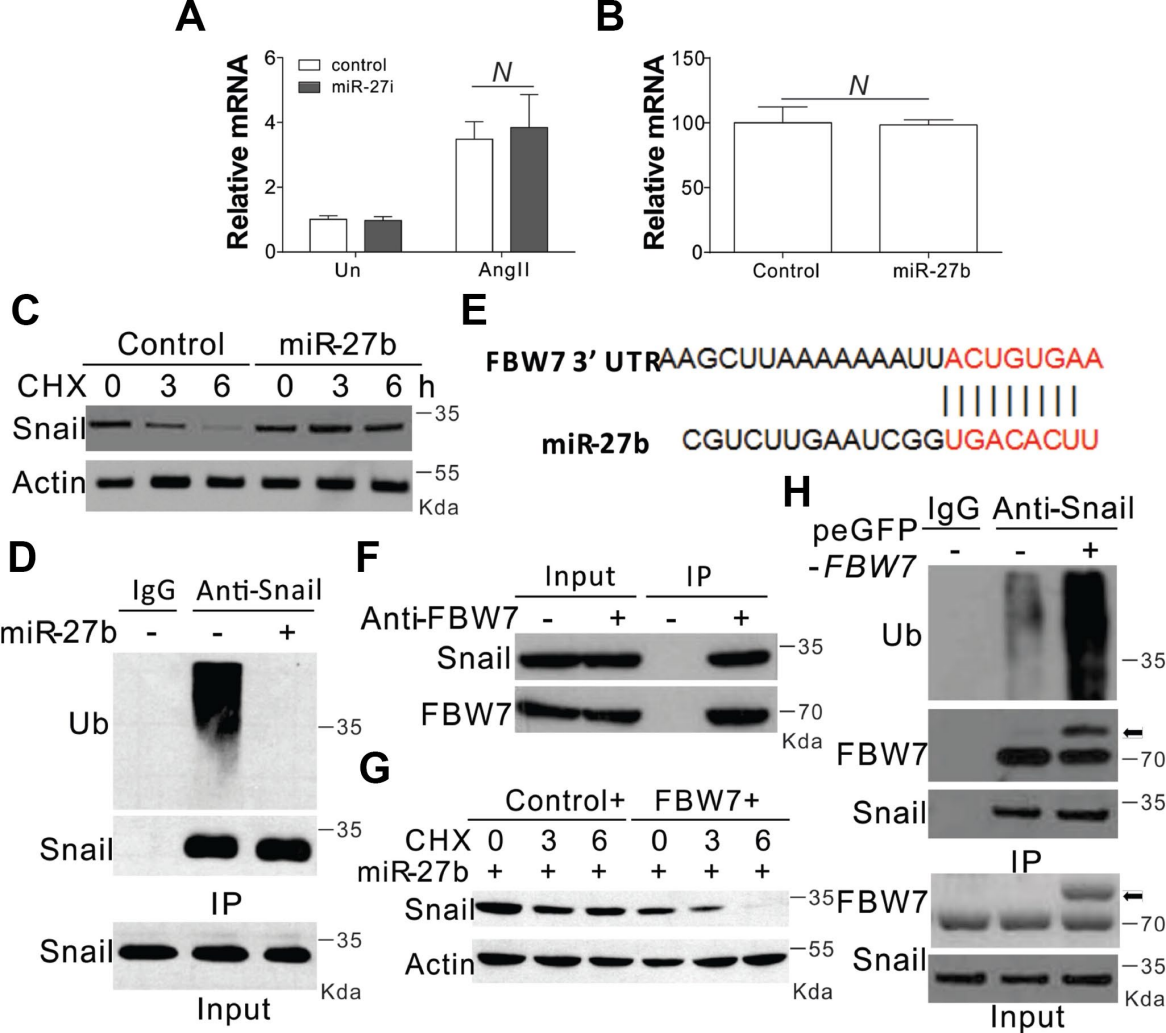
**miR-27b modulated the expression of Snail by inhibiting its degradation** (**A**, **B**) The mRNA level of Snail in CFs treated with AngII combined with miR-27i (**A**) or miR2-7b (**B**). (**C**) The effect of miR-27b on the expression of Snail in CFs treated with cycloheximide (CHX, 1 μg/ml) at indicated time points. (**D**) Roles of miR-27b in Snail ubiquitination. (**E**) Characterization of the messenger RNA (mRNA) of FBW7, which depicted miR-27b binding site (BS) in its 3′-untranslated region (UTR). (**F**) The interaction of FBW7 and Snail in CFs. (**G**) The effect of FBW7 overexpression on the expression of Snail in CFs treated with cycloheximide (CHX, 1 μg/ml) at indicated time points. (**H**) The effect of FBW7 overexpression on the ubiquitination of Snail. Arrow indicates the GFP-FBW7. Data were represented as mean ± SEM (n=4). N, *p*>0.05.

In the next step, we tried to explore the mechanism underlying miR-27b mediated induction of Snail. We firstly investigated the transcriptional control of Snail, and found that AngII substantially enhanced the mRNA levels of Snail (Figure 4A). However, the AngII mediated induction of Snail was clearly un-influenced in the presence of the miR-27b antagonist (Figure 4A). Supplementation of miR-27b in CFs also failed to show any obvious variation in the mRNA expression of Snail (Figure 4B). These results suggested that miR-27b did not target Snail at its transcriptional level. We therefore investigated the role of miR-27b in its protein expression. DNA translation inhibition by cycloheximide (CHX) led to degradation of Snail within 6 h (Figure 4C). However, miR-27b transfection in CFs inhibited the degradation of Snail (Figure 4C). The ubiquitination of Snail was also observed in CFs when pretreated with protease inhibitor (MG132, 5 μM) (Figure 4D). As expected, miR-27b supplementation also compromised the ubiquitination of Snail (Figure 4D), suggesting that miR-27b mediated the induction of Snail by inhibiting its ubiquitination and degradation.

The bioinformatics tool TargetScan (http://genes.mit.edu/targetscan/) was employed for the screening of the potential miR-27b target mRNAs involved in protein ubiquitination, so as to study the possible impact of miR-27b on Snail ubiquitination. FBW7 was identified in this process, where mRNA 3′-UTR regions were composed of seed sequences and flanking nucleotides matching miR-27b (Figure 4E). Next, we investigated the effect of FBW7 on the degradation and ubiquitination of Snail and found that in the absence of any treatment, FBW7 can interact with Snail in the CFs (Figure 4F). Enhanced expression of FBW7 in CFs abolished the protective effect of miR-27b on the degeneration of Snail (Figure 4G). Furthermore, overexpression of FBW7 also increased the ubiquitination of Snail (Figure 4H). Therefore, FBW7 was found to have an intermediary role in the Snail protein degradation in the CFs.

### FBW7 acts as the target of miR-27b in cardiac fibrosis

**Figure 5:**
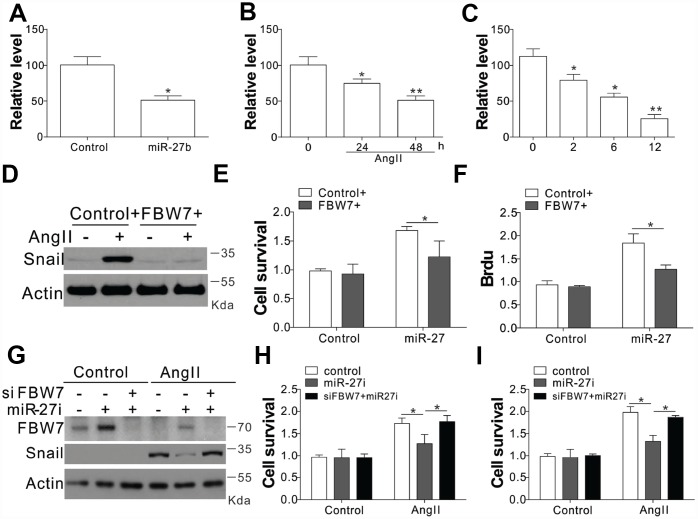
**FBW7 was the miR-27b target in cardiac fibrosis** (**A**–**C**) The mRNA levels of FBW7 in serum-treated CFs (**A**), AngII (**B**), or miR-27b (**C**). (**D**) Snail expression in CFs transfection with miR-27b and/or peGFP-FBW7 plasmids. (**E**, **F**) The proliferation of CFs treated in (**D**) were analyzed by MTT test (**E**), and BrdU assay (**F**). (**G**) CFs transfected with miR-27i and/or FBW7 siRNA were subjected to AngII treatment as indicated. Snail expression was analyzed using WB. (**H**, **I**) The proliferation of CFs treated in (**G**) was analyzed by MTT test (**H**), and BrdU assay (**I**). Data were represented as mean ± SEM (n=4). *, *p*<0.05; **, *p*<0.01.

In order to verify the role of FBW7 as the downstream target miR-27b, its mRNA levels were investigated under different circumstances of miR-27b activation. The transfection of miR-27b or treatment of AngII in the CFs substantially suppressed the transcript levels of FBW7 (Figure 5A, 5B). The mRNA expression of FBW7 was lowered at 2, 6, and 12 days post-CAL in the PIA of rats heart, when compared with sham-operated animals (Figure 5C). Consistently, miR-27b transfection or AngII treatment also suppressed the luciferase activity of FBW7 (Supplementary Figure 3A, 3B). Inhibition of miR-27b by its antagonist recovered the luciferase reporter activity (Supplementary Figure 3B). Therefore, these results collectively suggested that miR-27b directly targeted the 3’-UTR of FBW7, and suppressed its expression. We transfected the CFs with FBW7 plasmid along with miR-27b so as to verify the effect of FBW7 on the miR-27b mediated proliferation of CFs and matrix production. Our results indicated that FBW7 expression in CFs suppressed the induction of Snail, collagen I and III, and MMP-9, mediated by miR-27b transfection (Figure 5D). Consistently, FBW7 overexpression also suppressed the proliferative effect of miR-27b on CFs (Figure 5E, 5F). Furthermore, antagomiR-27b treatment rescued the expression of FBW7, which was hindered by AngII treatment, but suppressed the induction of Snail (Figure 5G). Silencing of FBW7 by its siRNA also abolished the suppressive effect of antagomiR-27b on the expression of Snail (Figure 5G), and promoted cell proliferation (Figure 5H, 5I). Cumulatively, our results suggest that miR-27b mediates the induction of Snail by targeting FBW7 regulation.

### Inhibition of miR-27b suppresses interstitial fibrosis of the infarcted heart

**Figure 6:**
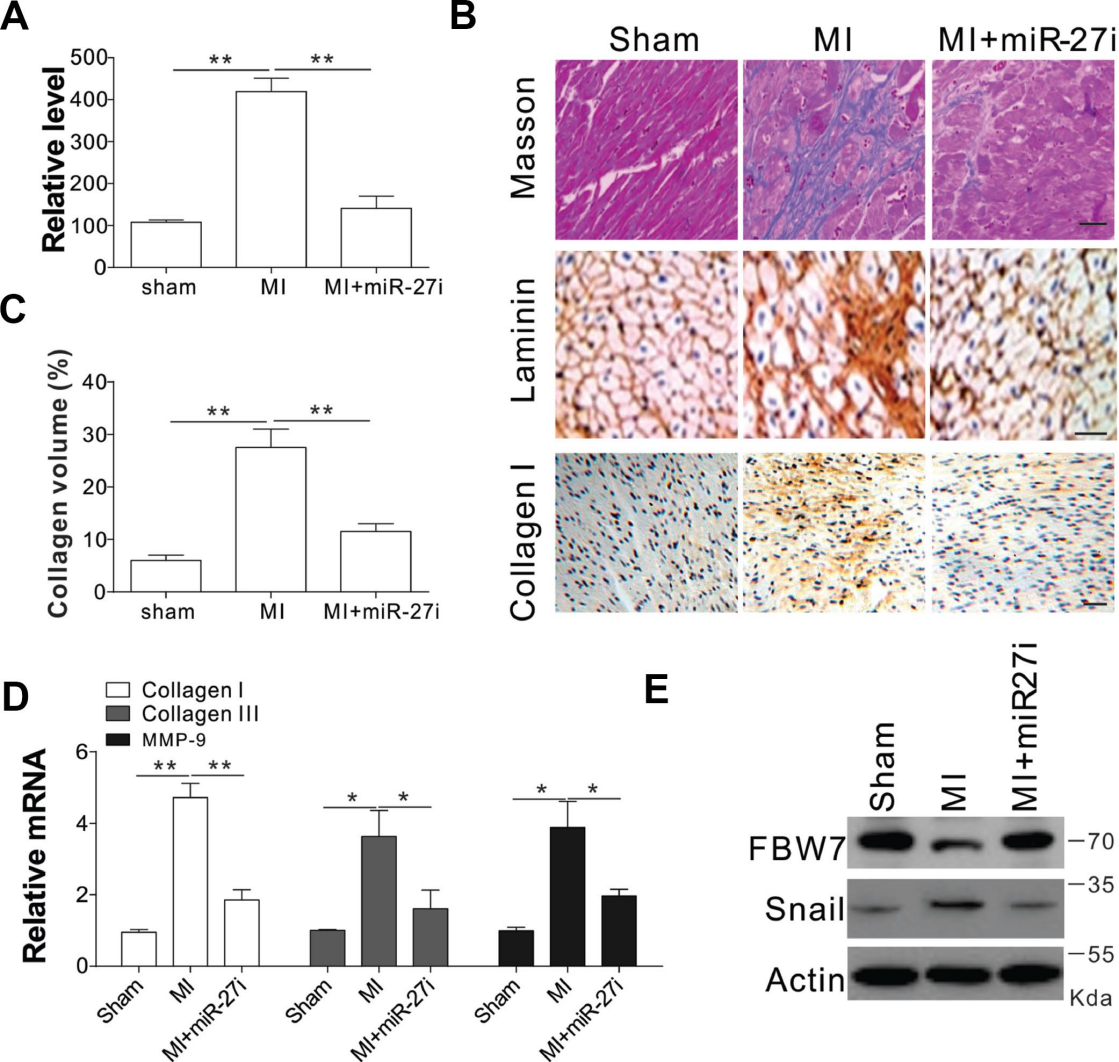
Antagomir-27b attenuated cardiac fibrosis in rat model of MI (**A**) Real-time PCR results of miR-27b levels in miR-27i or saline-treated specimens (3 weeks post-injection). (**B**, **C**) Analytical results of miR-27i-treated peri-infarct area of rat heart (3 weeks post-treatment). (**B**) Typical heart sections after treatments of Masson trichrome staining, laminin and collagen I immunostaining. Scale bar, 20 μm. (**C**) The percentage of tissue area represented the deposition of collagen I, where the automated image analyzer was used for its quantification. (**D**) Quantitative reverse transcription–PCR results of collagen I, collagen III, and MMP-9 mRNA levels. (**E**) FBW7 and Snail expression in rat heart. Data were represented as mean ± SEM (n=6). *, *p*<0.05; **, *p*<0.01.

To test whether miR-27b inhibition was truly beneficial for cardiac diseases, we injected antagomir-27b (miR-27i) into rat subjected to coronary artery ligation (CAL) induced myocardial infarction (MI). First, we tested the effect of antagomir-27b *in vivo* and found that mature miR-27b was significantly downregulated in hearts by antagomir-27b administration (Figure 6A). One of the main factors affecting the cardiac compliance MI depression and myocardial stiffness aggravation is interstitial fibrosis. Masson trichrome and Laminin staining were conducted on the tissue sections to evaluate the deposition of ECM. In comparison to the sham-operated control groups, the MI groups showed obviously elevated interstitial fibrotic area and collagen accumulation (Figure 6B, 6C). Administration of antagomir-27b significantly alleviated the collagen I deposition and the fibrotic area (Figure 6B, 6C). Additionally, the peri-infarct myocardium shows a reduction in collagen I, collagen III, and MMP-9, indicating that antagomir-27b mitigated the peri-infarct ECM deposition (Figure 6D). In addition, the variations of FBW7 and Snail expression in MI heart PIA were reversed upon antagomir-27b treatment (Figure 6E). Therefore, our *in vivo* data also supports the significant effect of miR-27b on cardiac fibrosis after MI.

## DISCUSSION

Cardiac fibrosis might be associated with unfavorable cardiovascular outcomes, since it plays a key role in cardiac remodeling [15]. As reported previously, overexpression of miR-27b in primary cultured cardiomyocytes promoted hypertrophic cell growth by targeting the peroxisome proliferator-activated receptor-γ (PPAR-γ) [11]. Nevertheless, no clear evidence was shown for the mechanism of miR-27b in cardiac fibrosis. To investigate the function of miR-27b in the cardiac fibrosis after cardiac infarct, CFs treated with AngII were used as an *in vitro* model of cardiac fibrosis [16], and experiments in rats CAL induced cardiac infarct were used as an *in vivo* model. Collagen generation and CF proliferation were enhanced post upregulation of miR-27b due to cardiac infarction. The present investigation showed that miR-27b inhibition impaired cardiac function of the MI hearts by suppressing the cardiac fibrosis in CAL heart. Additionally, miR-27b was observed to stimulate cardiac fibrosis via suppression of FBW7-mediated Snail degradation (Supplementary Figure 4).

The miRNA dysregulation is a commonly observed phenomenon in a wide range of diseases [17] and their pathogenesis. In ischemic heart disease, dilated cardiac myopathy, aortic stenosis, and many other heart-related diseases, the cardiac tissues often show decreased miR-101 expression [18]. Compared with CMs, CFs showed a higher abundance of miR-30c (antifibrotic miRNA) [19]. The levels of the profibrotic miRNA, miR-21, are increased selectively in fibroblasts of the failing heart [20]. In this study, we observed that miR-27b was upregulated in cardiac tissue after infarct. Furthermore, we provided the first direct evidence to prove that miR-27b overexpression is sufficient to induce cardiac fibrosis and proliferation both *in vitro* and *in vivo*. It is noteworthy that miR-27b is frequently upregulated in pressure-overloaded hypertrophic hearts [11, 21]. Nevertheless, no clear results have been achieved pertaining to the role exerted by miR-27b on cardiac fibrosis. Here, the loss-of-function and gain-of-function routes were employed to investigate the *in vivo and in vitro* impact on cardiac fibrosis, both pathologically and physiologically. Notably, the overloaded pressure-induced fibrosis effects were clearly abated by miR-27b inhibition *in vivo*, which is of clinical significance to these diseases.

The alteration in the key signaling pathways during the pathological process of fibrosis is the usual reason for the miRNA-mediated fibrotic regulation at the molecule level. Interestingly, miR-21 augments extracellular signal-regulated kinase–mitogen-activated protein kinase activity through inhibition of sprouty homolog 1 and thus regulates fibroblast survival and growth factor secretion [8]. Also, miR-30 and miR-133 directly participate in the downregulation of Hey inducer (connective tissue growth factor) ECM synthesis to exert control over the extent of interstitial fibrosis [19]. The present study demonstrates that miR-27b could target FBW7 and suppress Snail degradation, which promotes CF proliferation and ECM synthesis, resulting in cardiac fibrosis. In the mice group with carbon tetrachloride and ureteral obstruction treatments, the liver and kidney fibrosis are strongly associated with a zinc finger transcription factor (Snail) which is frequently seen to be expressed in the mesenchymal cells [22, 23]. In the heart, Snail has been shown to be expressed in cardiac fibroblasts and contribute to fibrosis, especially in mesenchymal (non-epithelial) cells in the heart following injury [24]. Snail is also involved in the expression of crosslinking genes and modifications in the extracellular collagen. Collagens I and III are the primary collagens secreted by the cardiac fibroblasts/myofibroblasts in the infarcted myocardium in response to pro-fibrotic factors. TGF-β, a potent stimulator of collagen production by cardiac fibroblasts, is induced in response to cardiovascular injury. It has been reported that antifibrosis treatment can be achieved by targeting TGF-β. However, due to the additional functions of TGF-β in different fibrotic pathologies such as post-MI remodeling (leading to heart failure progress) and postangioplasty restenosis, no evidence was obtained for its role in the cardiovascular system [1]. In contrast, Snail regulates the fibrosis pathway more specifically by promoting fibroblast proliferation and ECM production in fibrotic disorders. It was also reported that Snail absent cardiac fibroblasts have reduced collagen I mRNA expression and collagen deposition in response to TGF-β, suggesting that manipulating the expression of Snail is an alternative means to treat cardiac fibrosis. Our data indicated that miR-27b promoted the expression of Snail without the involvement of the TGF-β/SMAD pathway, which makes miR-27b inhibition to be a promising mode of treatment for cardiac fibrosis.

As to the mechanism of miR-27b mediated Snail expression, we found that FBW7 is the E3 ligase of Snail, which is targeted by miR-27b. It has been reported that miR-27 negatively controls the expression of the *FBW7*, which targets the cell cycle regulator, cyclin E [25]. Moreover, miR-27b is overexpressed and targets FBW7 in human hepatocellular carcinoma, which is associated with poor clinical outcome [26]. The overexpression of miR-27b led to obvious FBW7 suppression in CFs and infarcted hearts, proven by the high expression of miR-27b in infarcted hearts and proliferative CFs, and its potential key role in cardiac fibrosis. This also revealed the involvement of FBW7 in cardiac fibrosis. FBW7 could suppress tumors by its important function in cell-cycle progression, proliferation, and cell division in multiple cancers [27, 28]. FBW7 expression resulted in cell growth arrest, increased chemo-sensitivity, and inhibition of Epithelial-mesenchymal Transition (EMT), which led to suppressed lung cancer development [29]. Mechanically, FBW7 directly interacts with Snail, the transcription factor in EMT, and degrades its expression through ubiquitylation alternation in NSCLC [29, 30]. A number of previous reports have illustrated the role of FBW7 role in degrading Snail and enhancing EMT. However, no definite results have been acquired for their potential role in EMT progression of cardiac fibrosis. Our study revealed for the first time that FBW7 also contributes to inhibition of ECM formation in cardiac fibrosis by targeting Snail degradation.

To conclude, the present study succeeded in identifying miR-27b as a novel pro-fibrotic miRNA, raising an interesting prospect for its use as a potential candidate for targeted therapy. The infarcted hearts of rat showed alleviated fibrosis growth after miR-27b was inhibited i*n vivo*, where the FBW7/Snail pathway was inhibited. Therefore, antagomiR-27b can be exogenously applied for interceding cardiac fibrosis and its related pathological mechanisms.

## MATERIALS AND METHODS

### Blood samples of patients with cardiac fibrosis

We enrolled 19 patients with cardiac fibrosis and 16 healthy participants in the General Hospital of Tianjin Medical University. The inclusion criteria involved the diagnosis of cardiac fibrosis based on clinical information. Blood was collected from each of the participants for assessment.

### Animals and cardiac infarction model

Disease-free Sprague-Dawley (SD) male rats weighing ~200-250 g were utilized for our research and cultivated for one week beforehand in standard in-house conditions of 21 ± 1°C, humidity range of 55–60%, and free access to water and food. All experimental tests of our study were conducted in accordance with the rules of the Institutional Animal Care and Use Committee in the General Hospital of Tianjin Medical University, PR China. To induce myocardial infarction (MI), the rats were anesthetized with 100 mg/kg ketamine-xylazine and subsequently subjected to artificial respiration by inserting the polyethylene pipe via the mouth. The thoracotomy on the left part was conducted in the 4^th^ intercostal section. Meanwhile, the pericardium was left open to expose the heart. Next, the left descending artery (LAD) was ligated via a 5–0 silk thread, which led to infarct of the left ventricular free wall. Heart infarct was validated through significant S-T upregulation in ECG and defective cyanotic cardiac muscle. The control surgery constituted a fake stitch in the left cardiac pericardium. The rats got free access to water and food post-operation.

Modified antisense oligonucleotides (antagomir) were synthesized on order (GenePharma). Treatments were initiated 3 days post-surgery, and rats were injected with 0.2 ml saline, antagomir-27b, one shot a day, for three days through the tail veins [8].

### Isolated and cultivated CFs of newborn rats

Cardiac fibroblasts from newborn rats were processed as follows: over ten hearts extracted from SD rats aged 1–3 days were cut and immersed in 0.25% trypsin solution. The cell suspension was subjected to centrifugation and then resuspended in DMEM mixed with fetal bovine serum (10%), streptomycin (100 μg/ml), and penicillin (100 U/ml). The resuspension was plated onto culture flasks for 90 min, after which the fibroblasts were preferentially able to adhere to the flask bottom. This was followed by the removal of weakly adherent cells (or non-adherent cells), and media replacement. The cells were grown till confluency and passaged using trypsin. The incubation of cells was carried out with air (95%) and CO_2_ (5%) under the humidified condition at 37°C. In the FBS containing media, the cardiac fibroblasts (CFs; passage no. 2–4) were grown up to suboptimal confluency and used for experiments.

### Transfection of cells

The exponentially growing CFs were seeded into 6-well plates (2 × 10^5^ cells/well), followed by 24 h incubation. The transfection was performed using DharmaFECT 1 of Life Technologies, Germany, based on the manufacturer’s instructions. Firstly, in the Opti-MEM (Invitrogen, U.S.A.), the transfection agent was added to 75 nM mimics and incubated for 20 min, which was further mixed with the serum-free medium. After 1 day, hypertrophy-enhancing medium containing DMEM/high glucose, 1% Insulin-Transferrin-Selenium (ITS)+3, 50 μg/ml ascorbate-2-phosphate, 40 μg/ml L-proline, and 1 nM triiodothyronine (Sigma–Aldrich, U.S.A.) was used to replace the serum-free medium.

In the present study, miR-27b and its antisense oligonucleotides (antagomiR-27b) [31] were synthesized by GenePharma of Shanghai GenePharma Co, Ltd. The siRNA for FBW7 (L-115782-00-0005) and Snail (L-093687-02-0005) were purchased from ON-TARGETplus SMARTpool of Dharmacon (Lafayette, CO, USA). The negative control was a scrambled siRNA purchased from Dharmaco. The primer pair (forward, 5′-ccggtcgacatgacacaaaagtggacaaca -3′; reverse, 5′-tttcatgtcc acatcaaagtccaagcttgag-3′) was used for the amplification of FBW7 mRNA (XM_002729089) encoding the protein. This was followed by cloning the FBW7 cDNA sequence to the cytomegalovirus promoter-containing pEGFP-N1 vector, and the negative control was an empty pEGFP-N1 plasmid.

### Real-time PCR

Based on the manufacturer’s instructions, we isolated the total RNA including miRNAs from the cultured fibroblasts and cardiac tissues using NucleoSpin® miRNA kit (Macherey–Nagel, Germany). The miRCURY LNATM Universal RT microRNA PCR of Exiqon A/S, Vedbaek, Denmark, was used for reverse transcription. On an ABI 7500 fast real-time polymerase chain reaction system (Applied Biosystems, Foster City, CA), the RNA levels of FBW7, Snail, MMP-9, collagen I, and collagen III were detected using SYBR Green I incorporation method [18]. On the other hand, TaqMan MicroRNA Assay Kit (Applied Biosystems) was used to detect the miR-27b levels, where the internal control was U6. Additionally, PCR primers used for amplification of miR-27b are: 5′-TTTCTCGAGGAA GATGCTCACCAGCCCTTTA-3′ (miR-27b sense) and 5′-TTTTCTAGAGCATCATCTTGCCAGCGACT-3′ (miR-27b antisense) [11].

### Evaluation of cell viability

CytoTox 96^®^ Non-Radioactive Cytotoxicity Assay (lactate dehydrogenase; LDH; Promega Cor, Madison, WI, USA) was employed to assess the viability of CFs based on thiazolyl blue tetrazolium bromide containing 0.5 mg/ml MTT (Applichem Inc., Omaha, NE, USA) based on the manufacturer’s protocol.

### WB analysis

The total protein was extracted from the cultured CFs, and the levels were compared by immunoblotting the proteins based on their expression in the left ventricular peri-infarct area (PIA) of rats [32]. Antibodies used for probing were anti-FBW7, anti-Snail, anti-SMAD3/4, anti-phosphor-SMAD3/4 (Abcam, Cambridge, UK), anti-Twist, anti-TGF-β, anti-ubiquitin, and anti-actin antibody (Sigma-Aldrich Corp. St. Louis, MO, USA). The corresponding secondary antibodies were purchased from Stressgen Biotechnologies Corporation, Victoria, BC, Canada. Signals were used for detection using the Enhanced Chemiluminescence (ECL) kit (GE Healthcare, UK). Densitometry was employed to quantify the protein levels. β-actin was used as the loading control in the western blotting experiment for normalization.

### Masson’s trichrome staining and immunohistochemistry

Fixation was carried out for 24 h in phosphate-buffered saline (PBS) using formalin (10%). The cardiac tissues were dehydrated with alcohol, followed by paraffin (4%) embedding. HE and Masson’s trichrome staining was conducted on the sliced sections (5 μm) of the tissues. The extent of myocardial fibrosis was assessed by detecting the collagen volume fraction (CVF) using Image-Pro Plus software. Finally, the mean CVF values were obtained by blinding one investigator to the group assignment.

In the present study, a previously described method was used to perform immunohistochemical staining [11]. Probing with anti-collagen I and anti-collagen III antibodies (1:200 dilutions for both) were conducted on the tissue sections for 2 h at 37°C. This was followed by PBS washing for three times and the addition of the corresponding secondary antibody. After 2 h of incubation at 37°C, the samples were subjected to PBS washing prior to the addition of 3, 3′ Diaminobenzidine (DAB) for 5 min, followed by hematoxylin counterstaining. Then the graded series of alcohol was prepared for dehydration, and xylene was employed for stepping. Subsequently, the samples were mounted with neutral gums. An inverted microscope (Nikon Eclipse TS 100, Japan), was used for the observation of brown granules in the cells, with a random selection of 6 fields.

### Luciferase reporter assay

The 3′UTR of the *FBW7* luciferase reporter plasmid (pRL-TK 3′FBXW7 UTR) were obtained from Addgene (#26649). For the luciferase reporter assay, 293 cells were seeded in a 24-well plate and incubated for 24 hours before transfection. Next, luciferase constructs and miR-27b, or its antagonist were co-transfected into 293 cells using Lipofectamine 2000. Cells were collected at 48 hours after transfection, and measured using the Dual-Luciferase Reporter System (Promega, WI, USA), according to manufacturer’s protocols. Four independent experiments were performed, and data were presented as mean ± SD.

### Statistical analysis

The t-test was used for statistical analyses, along with the one-way analysis of variance (ANOVA). Additionally, the Bonferroniʼs test was conducted for pairwise multiple comparison. Data were represented as mean ± SEM, and a significant difference was considered when *P* < 0.05.

## Supplementary Material

Supplementary Figures
