# Supplementary material for: MicroRNA 27b promotes cardiac fibrosis by targeting the FBW7/Snail pathway
Source: Aging (Albany NY). 2019 Dec 23;11(24):11865–79. doi: 10.18632/aging.102465 (PMC6949061; doi:10.18632/aging.102465)
Supplement: Supplementary Figures [file aging-11-102465-s001..pdf]

SUPPLEMENTARY FIGURES

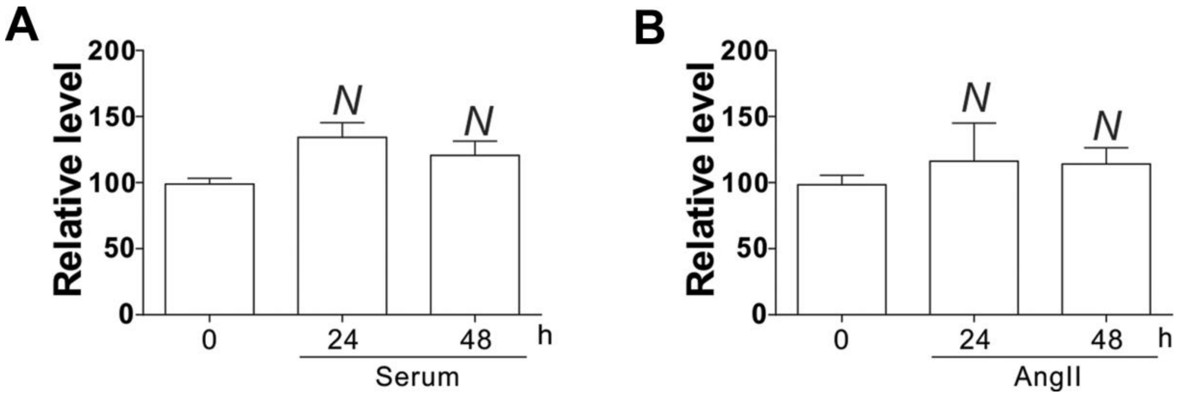

Supplementary Figure 1. The miR-27b expression in CMs. The miR-27b expression in CFs treated with 10 % serum of rats (**A**) or angiotensin II (AngII, 100 nM; **B**) at designated time points.

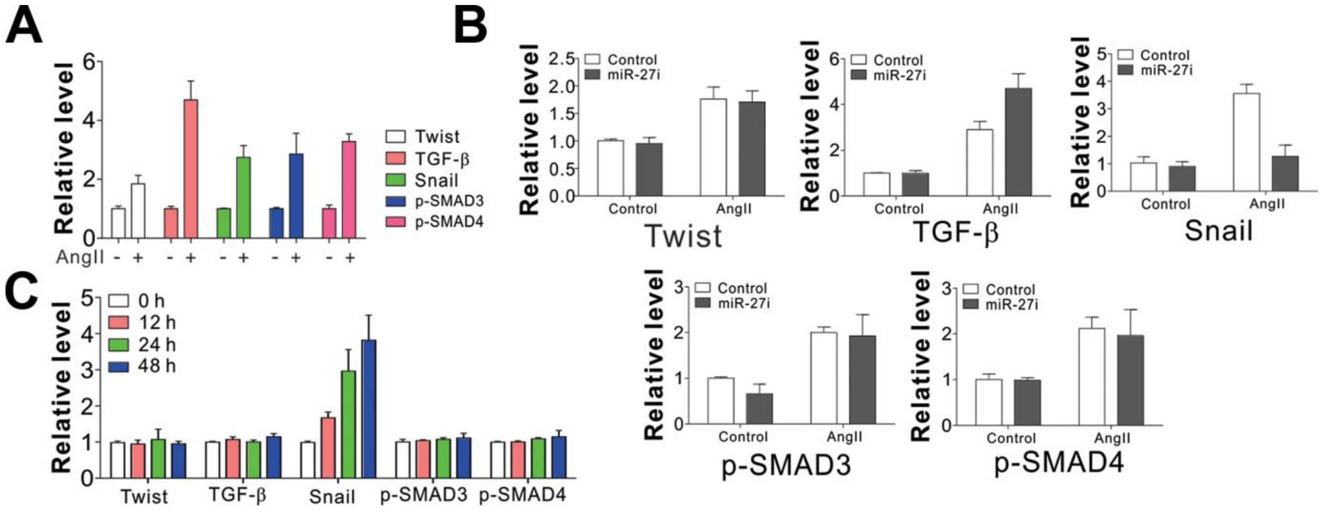

Supplementary Figure 2. The western blot densitometry of several proteins as shown in Figure 3A–3C.

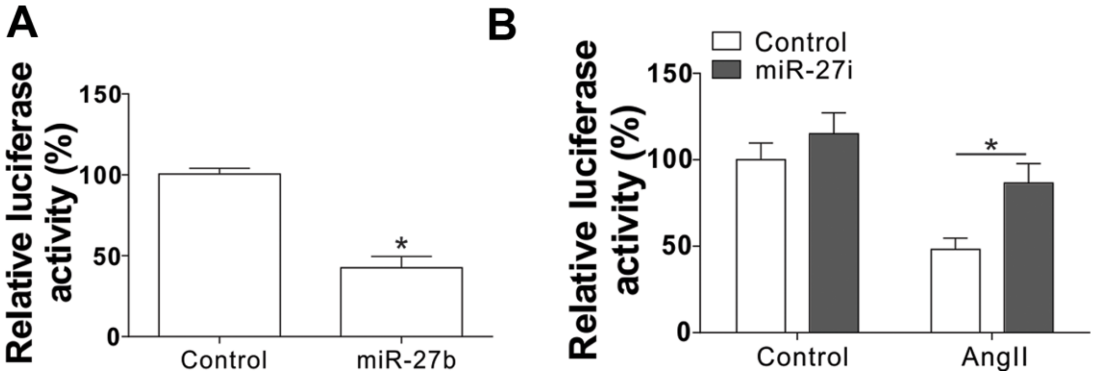

Supplementary Figure 3. miR-27b targets FBW7 3'-UTR. (**A**) The 3'-UTR of FBW7 luciferase reporter activity upon miR-27b co-transfection. (**B**) The 3'-UTR of FBW7 luciferase reporter activity upon AngII treatment with or without co-transfection of miR-27i.

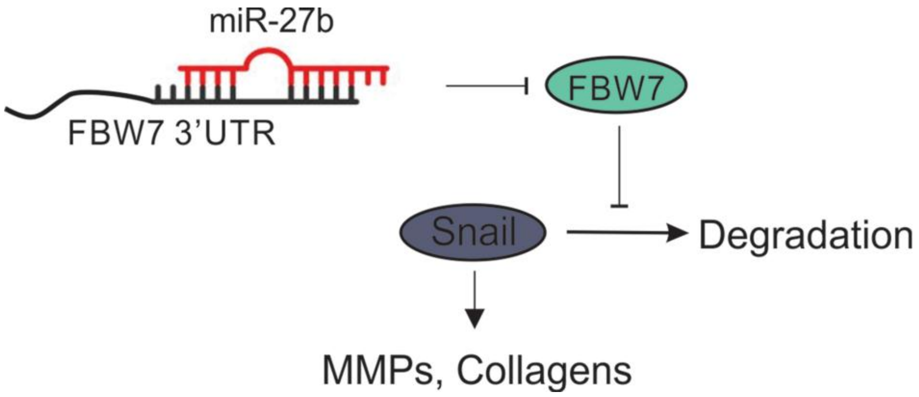

Supplementary Figure 4. A model of action.
